# Editorial: New analytical method developments for metallomics research

**DOI:** 10.3389/fchem.2022.1071474

**Published:** 2022-11-08

**Authors:** Bernhard Michalke, Tamara Garcia-Barrera, Volker Nischwitz, Nikolay Solovyev

**Affiliations:** ^1^ Research Unit Analytical BioGeoChemistry, Helmholtz Center München, Helmholtz Association of German Research Centres (HZ), Neuherberg, Germany; ^2^ Research Center of Natural Resources, Health and the Environment (RENSMA), Department of Chemistry, Faculty of Experimental Sciences, University of Huelva, Huelva, Spain; ^3^ Weihenstephan-Triesdorf University of Applied Sciences, Weidenbach, Germany; ^4^ Department of Life Sciences, ATU Sligo, Atlantic Technological University, Sligo, Ireland

**Keywords:** sample preparation, speciation, lipidomics, chromium speciation in *T. officinale*, anatagonistic interaction of selenium and cadmium, isotopic composition of redox-active elements, DNA interaction with suspended particulate matter

Metallomics research combines chemistry, and the biological, and/or environmental sciences, employing a quantitative system approach, and covers the fields of metalloproteomics, metallometabolomics, and ionics metal species. From an analytical chemistry viewpoint, metalloproteins, metallometabolites and ionic forms of elements with different valence states are all referred to as metal species.

In our Research Topic “*New Analytical Method Developments for Metallomics Research*,” we specifically compiled recent knowledge and timely analytical approaches from the metallomics field. The collected articles report exciting results based on the use of sophisticated techniques for metal speciation, spatial metal distribution in cells or focus on advanced approaches including species preserving sample preparation methods, being capable of redox speciation and subsequent metabolomics and lipidomics research. Finally, improved instrumental developments and quantification strategies based on isotope dilution procedures and high-precision isotopic analysis are reported.

The article of Blume et al. is methodically oriented and introduces investigations on a novel protocol for combined extraction of lipids and metal speciation.

The authors used *Caenorhabditis elegans* as a relevant animal model and focused on iron redox speciation and parallel lipid profiling. In this manuscript, the achieved extraction efficiencies were comparable to the reference method. This allows extracting the lipids of important classes at considerably reduced workload and lab-time. Further, the new protocol is capable of the simultaneous extraction of lipid species, free iron, and other metals simultaneously, while maintaining native redox conditions for metals. Thus, the quantification of free metal species, iron (II/III) ratio together with the lipidomic profile from the same sample was demonstrated successfully.

Marković et al., too, focused first on method development and applied their method then to speciation analysis and bio-imaging of chromium in dandelion plant (*Taraxacum officinale*).

They developed a new analytical Cr-speciation procedure in plants by HPLC-ICP-MS using a strong anion-exchange Mono Q column for Cr species separation. Their method provided column recoveries between 100 and 104% and high repeatability. Low limits of detection (<0.37 ng ml^−1^ Cr) were achieved. The designed method was applied to Cr speciation analysis in *T. officinale* grown in soil with a high or low Cr content to study the uptake and metabolism of Cr species. Cr was primarily detected in the roots in dandelions grown in Cr-rich soil or Cr-nitrate-treated plants. Contrarily, Cr was found to be distributed evenly in the roots and green parts of the plants treated with Cr(VI). The following Cr species were found in dandelion roots and leaves: Cr-aconitate, Cr-malate, and Cr-quinate. The study indicated Cr(VI) to be completely reduced and converted into Cr(III) complexes. Finally, LA-ICP-MS imaging showed primary localization of Cr in the apex of leaves for the plants grown in Cr-rich soil.

Ramirez-Acosta et al. aligned their research focus on the interaction of selenium and cadmium in human hepatic cells. The antagonistic relationship observed was shown to be facilitated by selenoproteins.

In their study, the authors aimed to evaluate particularly the antagonistic effect of selenomethionine (SeMet) *vs.* Cd toxicity in HepG2 cells. They cultured cells at different SeMet concentrations and determined endpoints by MTT assay and metabolic parameters, such as concentrations of glutathione peroxidase (GPX), selenoprotein P (SELENOP), selenoalbumin (SeAlb), and selenometabolites by using column switching and species-unspecific isotopic dilution ICP-MS. Technically, they used two-dimensional size exclusion and affinity chromatography coupled to ICP-QqQ-MS. Enhanced viability and diminished Cd accumulation in HepG2 cells was observed under Cd and SeMet co-exposure. Se-supplemented cells increased the levels of selenometabolites, GPX, SELENOP, and SeAlb, whereas Cd significantly reduced the concentration of selenometabolites and SELENOP. The authors conclude, that SeMet may alter the cellular Cd accumulation, whereas, Cd triggers the suppression of selenoprotein synthesis.

In a further exciting article, Hobin et al. performed high-precision isotopic analysis of Cu and Fe *via* MC-ICP-MS and find lipopolysaccharide (LPS)-induced inflammatory effects in blood plasma and brain tissues.

In this article, specifically, the trace element concentrations and the isotopic composition of redox-active essential elements (copper and iron) were studied in murine blood plasma and brain compartments (hippocampus, cortex, brain stem, and cerebellum) to investigate the alterations associated with sepsis-associated encephalopathy. Elemental analysis was accomplished with ICP-sf-MS, whereas MC-ICP-MS was employed for isotopic analysis. The highest Cu concentrations were reported in the cerebellum, which was the most prominent in the aged mice. Both redox-active elements (Cu and Fe) had a heterogeneous isotopic distribution within the mouse brain. Compared to controls, Cu was found to redistribute isotopically between hippocampus and cerebral cortex, as well as, between brain stem and hippocampus in the LPS-injected mice. The cerebellar Fe isotopic composition showed age dependence, being significantly lighter in the aged mice. However, in the aged LPS-injected mice, the authors found a shift to a heavier isotopic composition of Fe compared to controls. Finally, it was concluded that high-precision isotopic analysis provided additional information on metal biological activity not reflected in elemental concentrations.

In the last article of this research topic, Nischwitz et al. dealt with cascade filtration with PCR detection and field-flow-fractionation-ICP-MS.

They use these technologies for the characterization of DNA interaction with suspended particulate matter. Noteworthy, in the environment, a relevant number of antibiotic resistance genes (ARGs) are binding to particulate matter, which improves their stability and affects their transport and dissemination behavior. Therefore, this study aimed to establish a novel approach for the direct characterization of DNA particle interactions using cascade filtration and field-flow fractionation. The authors used a calf-thymus DNA-spiked surface-water sample as a model to investigate the following key primary method parameters: membrane composition, molecular weight cut-off, and carrier composition. Multi-element detection by ICP-MS enabled parallel monitoring of clay *via* the Al, Fe, and Si signals and that of DNA *via* the P signal. Matching peak profiles in the ARG and DNA-spiked water sample supported adduct formation. The applicability of the novel post-channel filtration approach designed was successfully illustrated by analyzing free calf-thymus DNA and clay–DNA adducts.

Summarizing, this short article collection nicely shows some of the new, exciting developments which may further advance the metallomics field in the future.

